# Nutritional care in metastatic RCC: patient experiences and reported unaddressed needs

**DOI:** 10.1007/s00520-025-09801-2

**Published:** 2025-08-04

**Authors:** Karin Kastrati, Jutta Huebner, Anna P. Kipp, Viktoria Mathies

**Affiliations:** 1 Klinik Für Innere Medizin II, University Hospital Jena, Am Klinikum 1, 07747 Jena, Germany; 2 Department of Nutritional Physiology, Institute of Nutritional Sciences, Friedrich Schiller University Jena, Jena, Germany

**Keywords:** Cancer, Kidney cancer, Renal cell carcinoma, Nutrition, Malnutrition, Side effects

## Abstract

**Purpose:**

Although renal cell carcinoma (RCC) presents unique nutritional challenges due to the disease itself and treatment side effects, little is known about the prevalence of nutritional issues among RCC patients in a real-world setting. This study aimed to investigate the patient-reported prevalence of nutritional issues and the response of healthcare teams to these challenges.

**Methods:**

A survey among RCC patients in Germany was developed in collaboration with patient organizations and included 46 questions covering demographics, nutritional issues, and cancer care experiences. It was distributed online from April to July 2022. Responses from 94 German RCC patients were analyzed using descriptive and inferential statistics.

**Results:**

Nutritional concerns were reported by 60.6% of participants, with diarrhea (23.4%), loss of appetite (21.3%), and nausea (20.2%) being the most common issues. Unintentional weight loss was reported by 49.4% of patients, but only 13.9% were referred to nutrition specialists. More than two-thirds reported a negative or extremely negative impact due to these problems on their physical condition and quality of life. Additionally, 67% of patients felt that their nutritional needs were not taken seriously by their healthcare teams. Most patients (84%) think that nutritional care should be part of routine cancer care.

**Conclusion:**

The findings reveal significant gaps in the nutritional care of RCC patients. Screenings and proactive assessments do not appear to be performed as suggested by nutritional guidelines. Thus, nutritional counseling and support are obviously still not integrated into real-world comprehensive oncological care.

## Introduction

Worldwide, cancer is one of the leading causes of death with close to 20 million new cases in 2022 and almost 10 million deaths. Additionally, the numbers of new cases are expected to significantly increase over the next years. Statistics suggest that approximately one in five men or women will develop cancer in their lifetime and that one in nine men and one in 12 women will eventually die due to the disease [1, 2]. Next to the more common cancer types such as lung, breast, colon, or prostate cancer, renal cell carcinoma (RCC) is the 14th most common cancer globally, affecting almost 435,000 people each year [1].

Cancer is a disease that changes the lives of patients drastically. Health issues caused by the disease itself or the side effects of cancer therapies can have a major impact on the patients’ quality of life and overall prognosis [1, 3, 4]. Among the many challenges associated with cancer and cancer therapies, nutrition-related problems such as a loss of appetite, nausea, or diarrhea are rather common. Unfortunately, if not treated in time, they can result in metabolic alterations, anorexia, and nutrient malabsorption which can lead to malnutrition. In general, the prevalence of malnutrition in cancer patients ranges from 20 to 70% depending on tumor location, stage, and age [5]. A recent study performed in a German hospital revealed that 56.8% of the patients had suspected or already existing malnutrition [6]. Worryingly, high rates of malnutrition not only negatively affect quality of life but also worsen treatment outcomes and survival rates of cancer patients [2, 7, 8]. Previous studies have shown that up to 20% of cancer patients die due to the consequences of malnutrition rather than the tumor itself [2].

Accordingly, nutritional support and nutritional counseling are important and need to be a cornerstone of comprehensive cancer care. The focus of nutritional interventions should be to optimize the patients’ nutritional status in order to improve treatment tolerance and enhance clinical outcomes [2, 9, 10]. Nutritional problems should be considered at diagnosis and throughout the whole disease trajectory. Tailored nutritional interventions have been shown to minimize treatment-related side effects, preserve muscle mass, and improve the patients’ functional status [2, 11–13]. However, despite the growing evidence on the importance of nutrition and existing guidelines on clinical nutrition in cancer care, the literature and patient reports suggest that there still remains a need for standardized strategies to implement nutritional care in the day to day treatment plan to not completely overlook patients in need for nutritional counseling or to miss the right time [14]. In a second step, this approach should be more specific addressing the individual nutritional needs of each cancer patient.

In our study, we focused on patients with renal cell carcinoma (RCC), the most common form of kidney cancer. These patients face nutritional challenges due to the specific nature of their disease and its treatments [15, 16]. RCC often requires (partial) nephrectomy, resulting in reduced renal function which can complicate nutritional management due to increased loss of micronutrients or changes in protein metabolism [17, 18]. The treatment landscape for advanced or metastatic renal cell carcinoma (mRCC) includes a variety of options, such as monotherapies or combinations of immunotherapies (immune checkpoint inhibitors) and targeted therapies (tyrosine kinase inhibitors) [17–19]. All these therapies need to be administered in a long-term approach as long as they are effective and they often result in side effects that have a negative impact on food intake and the nutritional status of patients [15, 16, 20]. Therefore, patients frequently experience weight and muscle loss as well as metabolic abnormalities [15, 17, 21].

Despite these known challenges, data on the patient-reported impact of systemic treatments on everyday nutrition and on the reaction of healthcare providers to their nutritional problems remains limited. To address this knowledge gap, we conducted a survey among metastatic RCC patients currently undergoing therapy to evaluate their experiences and document patient-reported insights.

## Methods

To conceptualize the questions for the quantitative survey, qualitative interviews were conducted with members of the Nierenkrebs-Netzwerk Deutschland e.V. (Kidney Cancer Network Germany), a patient-driven non-profit support group for patients with RCC. An interview guide was developed for the qualitative patient survey. Patients were interviewed in semi-structured individual interviews via video call using Red Connect (KV-certified). The interview was divided into two main parts, each focusing on different aspects. In the first part, general questions regarding the patients’ experiences with side effects of the cancer treatment were discussed. The second part focused on side effects related to nutrition. Seven patients with metastatic renal cell carcinoma were interviewed from April to June 2021.

The answers to the qualitative interviews formed the basis for the quantitative survey. The online survey was conducted among patients with metastatic renal cell carcinoma using a newly developed questionnaire which had a mixture of open and closed questions, including social, clinical, and nutritional topics, based on the findings from the qualitative interviews conducted beforehand. The survey was developed together with the patient organizations Kidney Cancer Network Germany, the European Cancer Patient Coalition (ECPC), the European Nutrition for Health Alliance (ENHA), and researchers from Sapienza University of Rome and the University Hospital Jena. Before circulation, the questionnaire was tested by 10 patients from the Kidney Cancer Network Germany. No concerns were reported.

The questionnaire was circulated online via European patient organizations affiliated with ECPC. Data was collected from April to July 2022 via SoSci Survey. Patients were informed about the anonymity of the data and data protection laws were respected. The survey was approved by the ethics commission of the University Hospital Jena (Number of the ethical vote: 2022–2548 Bef).

The final questionnaire consisted of 46 questions separated into seven sub-categories:

1. Demographics
2. Questions related to the cancer journey
3. Questions related to the nutritional issues experienced
4. Responses of the cancer care team to nutritional issues
5. Questions relating to weight
6. Access and timing of nutritional care
7. Importance of nutritional care for patients during their cancer journey

The data from the questionnaires was transferred to IBM SPSS Statistics 28. The results were reported as median, mean, and standard deviation for the quantitative variables and frequencies and percentages for the categorical variables. Correlations were tested to compare the quantitative variables using the chi-square and *t*-test. The tests were considered statistically significant if *p* < 0.05.

For this publication, only data from German patients with renal cell carcinoma was analyzed.

## Results

### Demographic data and cancer journey

Of the 94 people with metastatic renal cell carcinoma living in Germany who completed the questionnaire, 76 (80.9%) reported that they were currently undergoing treatment while 18 (19.1%) were taking a break from therapy. The median age was 62 and two-thirds of the participants were male (*n* = 61, 64.9%) and 33 female (35.1%). More than half stated that they were retired (54, 57.5%) versus 27 (28.7%) who were still working, with 3 (3.2%) on sick leave. Further characteristics of the study group are shown in Table 1.

**Table 1:** Characteristics of the study group (n = 94) of patients with mRCC

	*n* (%)
*Age*	94 (100)
< 50	12 (12.8)
50–59	24 (25.5)
60–69	38 (40.4)
70 +	20 (21.3)
Median (min–max)	62 (40–82)
*Gender*	94 (100)
Male	61 (64.9)
Female	33 (35.1)
*Years from diagnosis*	94 (100)
0–2 years	26 (27.7)
3–5 years	22 (23.4)
6–8 years	19 (20.2)
> 8 years	27 (28.7)
*Level of education*	94 (100)
None or basic education	8 (8.5)
High school	32 (34.0)
University degree	50 (53.2)
Prefer not to say	4 (4.3)
*Area of living*	94 (100)
Rural	26 (27.7)
Suburban	24 (25.5)
Urban	43 (45.7)
I do not wish to say	1 (1.1)
*Current working status*	94 (100)
Employed	27 (28.7)
Self-employed	5 (5.3)
Unemployed and able to work	2 (2.1)
Unemployed and not able to work	2 (2.1)
Retired	54 (57.5)
On sick leave	3 (3.2)
Other	1 (1.1)
***Therapy***	94 (100)
Currently under treatment	76 (80.9)
Taking a break	18 (19.1)
*Type of current treatment (multiple answers possible)*	76 (100)
Chemotherapy	5 (6.6)
Targeted therapy (TT)	29 (38.2)
Immunotherapy (IO)	21 (27.6)
TT + IO combination	7 (9.2)
Radiotherapy	2 (2.6)
Question not answered	5 (6.6)
*Comorbidities before treatment*	***94 (100)***
Yes	34 (36.2)
No	58 (61.7)
Prefer not to say	2 (2.1)

### Nutritional issues

Nutritional issues are of great importance to mRCC patients. Fifty-seven participants (60.6%) stated that they were concerned about food and nutrition, that they were unsure about which foods to eat or not to eat, or how best to deal with nutritional issues. Interestingly, only 34 participants (36.2%) have experienced eating or nutritional problems, indicating that 24% of the patients were worried about nutrition without personally having nutritional problems. Thirty-four patients (36.2%) reported having comorbidities before treatment. From these patients, 16 (47.1%) also encountered nutrition-related problems. Therefore, nutritional issues could be comorbidity-induced as well as being treatment-related.

The patients reporting having experienced eating or nutritional problems (*n* = 34) described the most common problems as follows: diarrhea (22 patients, 64.7%), loss of appetite (21, 61.8%), nausea (19, 55.9%), food aversion (19, 55.9%), and pain (12, 35.3%). Other problems characteristic for chemotherapy such as vomiting and swallowing issues were less common in mRCC, with only 5 (14.7%) and 6 (17.6%) patients reporting having these problems. Interestingly, male participants appeared to encounter food aversion more often than female participants (12, 35.3% vs. 7, 20.6%), while women were reporting pain more often than men (8, 23.5% vs. 4, 11.8%). In our survey, binge eating occurred rather seldom (*n* = 5, 14.7%) and in women only.

**Fig. 1:**
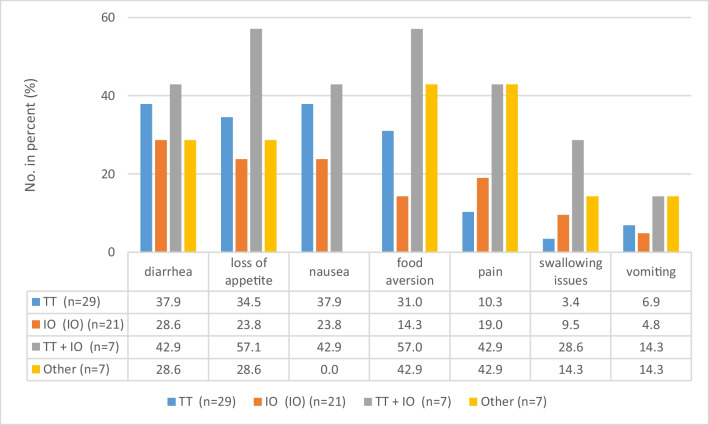
Nutritional problems reported according to type of treatment. TT, targeted therapy; IO, immunotherapy; numbers in percentage with* n*, number of patients receiving corresponding treatment set as 100%

The patient group undergoing targeted therapy (*n* = 29) reported diarrhea (11 patients, 37.9%) and nausea (11, 37.9%) as the most common problems. Patients receiving immunotherapy mentioned diarrhea (6 patients, 28,6%), loss of appetite (5, 23.8%), and nausea (5, 23.8%) as the most prevalent concerns. Further details are shown in Fig. 1.

**Fig. 2:**
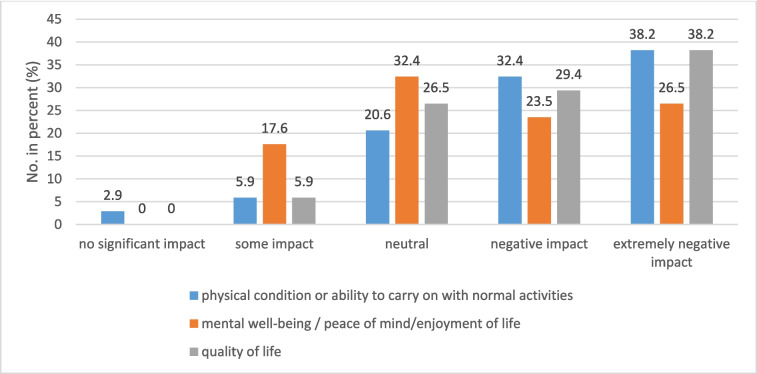
Impact of nutritional problems on physical condition, mental well-being, and quality of life. Numbers in percentage with *n* = 34 set as 100%

Thirty-four participants answered the question regarding the impact of these nutritional problems on their everyday lives. More than two-thirds (70.6%) stated that the impact on their physical condition or ability to carry on with normal activities was negative or extremely negative, with almost the same numbers for the negative or extremely negative impact of nutritional problems on quality of life (67.6%). Fifty percent (50%) reported a negative or extremely negative impact on their mental well-being. Almost nobody had experienced no impact of nutritional problems at all (Fig. 2).

**Fig. 3:**
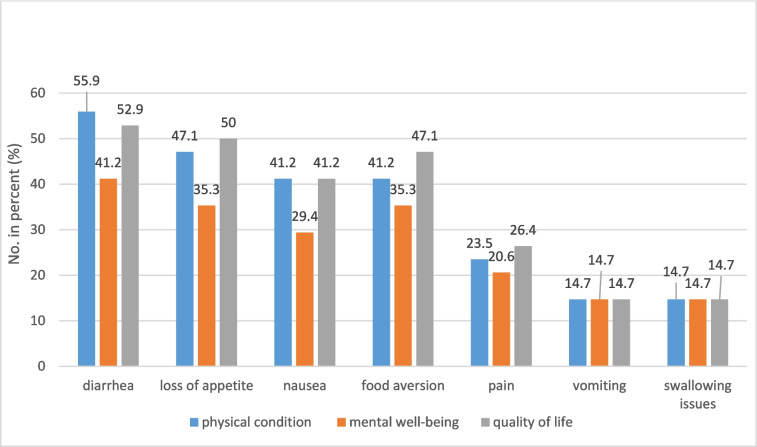
Nutritional problems with negative and extremely negative impacts on patients’ physical condition, well-being, or quality of life. Numbers in percentage with *n* = 34 set as 100%

Looking at the described nutritional issues of those patients who reported negative or extremely negative impacts due to nutritional problems on their physical condition, well-being, or quality of life, we could see that diarrhea had the most negative impact on all three aspects, followed by loss of appetite, nausea, and food aversion (Fig. 3).

### Response of the cancer care team to nutritional issues

Sixty-seven patients (74.4%) reported that their care team rarely or never asked them about nutritional problems. Only 3 (3.3%) stated that they were always asked or nearly asked every time they saw the care team. Regarding body weight check, 49 (54.4%) reported “rarely” or “never,” while 20 (22%) answered “always.”

Forty-three (45.7%) patients reported weight loss following diagnosis, with 35 out of the 43 (81.4%) stating that the weight loss was unintentional. Surprisingly, 29 of the 43 patients (67.4%) reported that they were not told that unintentional weight loss could interfere with their anticancer treatment and might reduce the outcome. The same number of participants (29, 67.4%) were not referred to a nutrition specialist (physician and/or dietitian) for advice/treatment regarding their weight loss. Only 6 participants reporting weight loss (13.9%) received nutritional counseling.

About two-thirds of all participants (57, 60.6%) reported a feeling of reduced force in their muscles. While 65 patients (69.1%) stated that they had not been informed about the importance of preventing cancer-related weight and/or muscle loss, 23 patients (24.5%) stated that they had been informed (the remaining 6 patients did not answer the question).

### Experience in getting dietary or nutritional advice

Regarding experience in getting advice on dietary or nutritional problems, 38 (40.4%) of the participants stated that they had difficulties while 52 (55.3%) reported no difficulties in that matter. Additionally, 63 (67.0%) indicated that they think that the care team does not take nutrition or nutritional issues seriously. Not surprisingly, 35 patients reported seeking advice from other sources. According to the patients, these included verified sources (e.g., cancer associations) on the internet (25, 71.4%), unverified online sources such as social media (5, 5.3%), and other sources such as books (16, 17.0%).

Forty-three (45.7%) participants believe that having better or earlier access to nutrition information or support (e.g., nutritional assessment, advice, nutrition supplementation) would have helped them on their cancer journey, 28 (29.8%) think that this was not the case. Additionally 79 patients (84.0%) reported that they believe that nutritional care (advice, support, or treatment) should be part of the routine, high-quality cancer care for all patients, only 4 patients (4.3%) disagreed. While 69 patients (53.5%) stated that in their opinion, nutrition was not given sufficient priority or attention as part of their cancer care, and 18 (19.1%) did not agree.

## Discussion

This study aimed to investigate the patient-reported prevalence of nutritional issues among mRCC patients in Germany and the response of their healthcare teams to these challenges. Although the number of participants in our survey was small, the demographics of the 94 participants were typical for mRCC patients and thus seem to represent the average mRCC patient rather well [1].

The data clearly shows that patients with mRCC often face nutritional problems which are not addressed or do not receive the attention that patients would like. While two-thirds of the participants (60.6%) were reporting concerns about food and nutrition and what foods to eat or how to deal with nutritional issues, one-third of patients (36.2%) stated that they encountered nutrition-related treatment side effects such as diarrhea, loss of appetite, nausea, and food aversion. This discrepancy between the proportion of participants expressing concerns about food and nutrition and those actually reporting nutrition-related treatment side effects could be due to several factors. One could be the lack of nutritional health literacy and information gaps regarding nutrition among patients. The psychological impact of a cancer diagnosis often leads to an increased urge to adopt a healthier lifestyle, including a healthier diet. In addition, patients often believe that adequate nutrition is important to help their immune system fight cancer and prevent disease progression [22]. However, as our survey revealed, in most cases, patients do not receive structural nutritional advice or information about their diet from their medical team and are therefore unsure about what they can or should eat.

More than two-thirds (70.6%) of the patients in our survey also reported that nutritional problems had a negative or extremely negative impact on their physical condition or ability to carry on with normal activities. Almost the same number (67.6%) reported that nutrition-related problems had a negative impact on their quality of life and every second participant saw a negative impact on their mental well-being (50%). In our survey, diarrhea seems to have the most negative impact on physical condition (55.9%), followed by well-being (41.2%) and quality of life (52.9%). In 2020, Chen et al. reported reduced physical functioning and health-related quality of life in breast cancer patients with heavy diarrhea [23]. The ESMO clinical practice guidelines mention diarrhea as one of the symptoms with the highest impact on health-related quality of life [24]. This is alarming as studies have also shown that quality of life is crucial for cancer patients [25], due to having an impact on treatment outcomes and survival [2, 7, 8]. Given the fact that mRCC treatment needs to be administered life-long and therefore side effects also persist, it is obvious that patients need a good side effect management to reduce the burden of these symptoms to improve their quality of life. Thus, it is essential that patients report treatment side effects as soon as they occur and that healthcare providers proactively ask whether there are problems.

Although the participants in our survey seemed to be struggling with nutritional problems that impact everyday life, a high number stated that these problems are not getting enough attention from healthcare providers. Unfortunately, so far, weight assessments and screening for nutritional problems and/or malnutrition are not mentioned in the guidelines on the treatment of renal cell carcinoma [17, 26, 27]. Suggestions to perform these assessments are only given in nutritional guidelines [2]. Although these guidelines suggest that all cancer patients should be screened for weight loss and malnutrition, it appears that this is not implemented in a real-world setting. The study by Trujillo et al. revealed that in the USA, only 53% of outpatient cancer centers reported screening for malnutrition [24]. Another survey from 2020 performed in Italy found that nutritional assessment at diagnosis was performed by only 27% of oncologists, with 16% using validated assessment tools [25]. With three out of four patients reporting that their care team rarely or never asked them about nutritional problems, we saw the same in our dataset. More than half of the participants reported that their weight was rarely or never checked. Although the literature clearly indicates that weight and muscle loss need to be prevented, there seems to be a lack of guidance towards patients [2, 28]. Also in our study, many patients reported that they were neither informed about the risks of unintentional weight and muscle loss during therapy nor about the importance of preventing cancer-related cachexia. Given the established link between cachexia, treatment tolerance, and survival, this represents a critical gap in mRCC management.

Obviously, further strategies are needed to implement existing guidelines. It would definitely help to embed a nutritional assessment in the treatment guidelines such as the ESMO treatment guidelines or the national German S3 guidelines for mRCC management. Further training for healthcare providers, especially urologists and oncologists, who are treating RCC patients regarding malnutrition and its consequences should be implemented, as well as easy-to-use assessment tools such as the Patient-Generated Subjective Global Assessment Short Form (PG-SGA SF).

Despite the high prevalence of nutritional problems, we could see that only a small number of participants reported receiving adequate support or advice from their healthcare teams. The high number of patients reporting not being referred to nutritional specialists even in cases of weight loss is particularly alarming, as early intervention is important in mitigating the progression of malnutrition and muscle loss. The low referral rates to nutrition specialists (13.9%) and the reliance on patients seeking advice through external sources underscore the systemic barriers to effective nutritional care. This seems to be true not only for RCC but all cancer types. Baguley et al. reported that overall, only 23% of patients received nutrition information from a dietitian after their cancer treatment and Eglseer et al. speak about a total dietitian referral rate of 16.8% [29, 30].

The fact that many patients relied on unverified online sources indicates an unmet need for accessible, evidence-based nutritional information. The lack of guidance not only risks misinformation but also underscores the need for healthcare providers to take a more active role in delivering nutritional care. This is even more important given the low health literacy among the German population. Schaeffer et al. reported that 58.8% of the participants in their health literacy study had low health literacy, with 48.3% reporting difficulties accessing information, while 47.7% had problems understanding and 53.5% had trouble applying the information [31].

Most participants in our survey (84.0%) stated that they would like to see nutritional care as an integral part of high-quality cancer care. Almost every second patient (45.7%) indicated that better or earlier access to nutritional support could have positively influenced their cancer journey. These findings highlight the need for changes to the nutritional care within oncology.

## Limitations

The study design of online research promoted within a self-help group clearly has limitations. In addition, we were looking at a rather small study group (*n* = 94) focusing on a single country, which might be limiting the generalizability. It seems likely that more patients who are familiar with web-based tools returned the online questionnaire. Accordingly, we cannot say whether the results are representative of all members of the Kidney Cancer Network Germany or patients with kidney cancer. It might also be likely that patients with advanced disease suffering from treatment side effects were contacting support groups more; thus, they were more likely to be included in the sample which again may mean that it is unrepresentative of the wider mRCC population. As this was a one-time questionnaire, a selection bias might be assumed since the accrued patients may be those who might already be experiencing nutritional problems such as weight loss or might be more warned of their nutritional status.

## Conclusion

Our study revealed the importance of nutrition for patients with mRCC, particularly given the unique challenges they face due to post-nephrectomy metabolic changes and nutrition-related side effects. It also highlights patient-reported gaps in nutritional screening and support within German cancer care routines. The findings emphasize the need for more proactive nutritional assessments, better communication between patients and healthcare teams, and the inclusion of nutritional care as a standard part of comprehensive RCC care. Addressing these gaps can not only improve treatment outcomes and overall patient satisfaction but also enhance the patients’ quality of life and mental well-being. Future studies should explore all possibilities to better implement nutritional screening tools, alongside training programs to enhance the awareness of healthcare providers regarding the importance of nutrition and the patients’ nutrition-related health literacy.

## Data Availability

To protect the privacy of the participating patients data will not be shared openly, but can be accessed upon request.
